# Factors Influencing the Serum Uric Acid in Gout with Cerebral Infarction

**DOI:** 10.1155/2021/5523490

**Published:** 2021-07-12

**Authors:** Yi Li, Hongyi Yang, Yao Tian, Lihua Duan

**Affiliations:** ^1^Department of Rheumatology and Clinical Immunology, Jiangxi Provincial People's Hospital Affiliated to Nanchang University, Nanchang, China; ^2^Jiangxi University of Chinese Medicine, Nanchang, China

## Abstract

**Background:**

Although the relationship between gout and cardiovascular has been well demonstrated, there is little information about the difference between gout with cerebrovascular disease and cardiovascular disease. In this study, the differences between gout with cerebral infarction (gout+CI) and gout with coronary heart disease (gout+CHD) and related factors that affect serum uric acid (sUA) levels in gout+CI were investigated by a cross-sectional study.

**Method:**

The patients from Jiangxi Provincial People's Hospital with gout+CHD, gout+CI, and gout with coronary heart disease and cerebral infarction (gout+CHD+CI) between 2016 and 2020 were included in this study, and the medical record data were collected and analyzed.

**Results:**

We observed significant differences in age, drinking, hypertension, long-term use of diuretics and NSAIDs, sUA, CRE, and blood glucose in patients with gout+CHD and gout+CI. The sUA level was significantly positively correlated with smoking, CRE, and TG in the gout+CI group and was only positively correlated with CRE in the gout+CHD group and the gout+CHD+CI group (*p* < 0.05). Interestingly, the sUA level was only negatively correlated with the age and gender in the gout+CI group (*p* < 0.05). After excluding factors with no significant statistical effect, only age, gender, smoking, CRE, and TG were included in the multiple linear regression model. It suggested that smoking, CRE, and TG are positively correlated with the sUA level, while age was negatively correlated with the sUA level.

**Conclusions:**

There are many discrepancies in clinical characteristics between gout+CHD patients and gout+CI patients, especially that the factors that affect UA levels are significantly different. The data also suggested that uric acid-lowering therapy may need to be strengthened in the young gout+CI patients with a history of smoking.

## 1. Introduction

Gout is a chronic inflammatory disease, which was caused by abnormal purine metabolism and decreased uric acid excretion leading to increased levels of sUA. It is characterized by swollen, hot, and painful joints. However, gout is a systemic disease, usually complicated by cardiovascular diseases, hypertension, dyslipidemia, diabetes, and other diseases [1]. Hyperuricemia is associated with an increased risk of cardiovascular disease [2]. Patients with gout have a higher risk of coronary heart disease than those without gout. In addition, compared with gout patients who have received antigout treatment, gout patients who have not received antigout treatment have a significantly increased risk of coronary heart disease [3]. However, there are few data on the relationship between gout and cerebrovascular diseases. A previous study has shown that higher sUA levels are an independent risk factor for coronary heart disease. In addition, a positive correlation between the risk of coronary heart disease and the level of UA was observed [4]. It has been reported that different UA concentrations have different effects on brain injury. An appropriate concentration of UA can significantly improve cell viability and reduce cell apoptosis and has a neuroprotective effect, while a high concentration of UA reduces cell viability [5]. Although increased levels of sUA have been shown to be associated with increased risk of cerebral infarction (CI) [6, 7], there are few data on the influencing factors of sUA levels in patients with gout and CI. Therefore, in this study, we conducted a cross-sectional study on the gout+CHD group, the gout+CI group, and the gout+CHD+CI patients to explore the characteristics of gout+CI patients and the factors that affect uric acid in these patients. The data showed significant differences in age, drinking, hypertension, long-term use of diuretics and NSAIDs, sUA, CRE, and blood glucose in patients with gout+CHD and gout+CI. In addition, there are many discrepancies in factors affecting sUA levels between gout+CHD patients and gout+CI patients, and the data also suggested that UA-lowering therapy may need to be strengthened in the young gout+CI patients with a history of smoking.

## 2. Materials and Method

### 2.1. Research Objects

All patients who were diagnosed with gout complicated with CHD, gout complicated with CI, or gout complicated with CHD and CI and then discharged from Jiangxi Provincial People's Hospital from January 1, 2016, to November 1, 2020, were included in the cross-sectional study. Criteria included a discharge diagnosis of gout+CHD, gout+CI, or gout+CHD+CI and age ≥ 18 years. Criteria excluded anemia, chronic tumor, myelodysplastic or lymphatic proliferative disease, primary renal inadequacy, secondary gout, chronic hepatic insufficiency, pregnancy or lactation in females, and patients with incomplete information. This study was approved by the ethics committee of Jiangxi Provincial People's Hospital. Patients' information was acquired and collected from the electronic medical record system of Jiangxi Provincial People's Hospital, without actual contact with any patients.

The main data sources for the diagnosis of gout, CHD, CI, and hypertension were acquired from the hospital electronic medical record, especially the discharged diagnosis and medical history, which includes the usage records of maintenance drugs (e.g., uric acid-lowering drugs and antihypertensive drugs) and CHD medicine treatment for secondary prevention, and previous medical records. We collected demographic characteristics, clinical characteristics, and laboratory parameters (e.g., age, gender, BMI, blood pressure, smoking, drinking, sUA levels, creatinine, total cholesterol, triglyceride, low-density lipoprotein, blood glucose, and long-term use of diuretics and nonsteroidal anti-inflammatory drugs) from the hospital's discharge record. Fasting venous blood was collected in the morning for all laboratory tests.

### 2.2. Diagnostic Criteria

*Gout*: patients who had a diagnosis of gout and had received drugs to lower sUA before admission, such as febuxostat, colchicine, or allopurinol according to the previous medical history, or patients who were newly diagnosed with gout during their hospitalization between 2016 and 2020, which met the 2015 ACR/EULAR classification criteria for gout [8].

*CHD*: patients who had a diagnosis of CHD and had received secondary prevention for CHD before admission according to the previous medical history or patients who were newly diagnosed with CHD on the basis of the typical clinical symptoms, electrocardiogram characteristics and myocardial necrosis markers, or coronary angiography, during their hospitalization from January 1, 2016, to November 1, 2020.

*CI*: patients who had a diagnosis of CI and had received lipid-lowering, antiplatelet aggregation and other relevant treatment before admission according to the previous medical history or patients who were newly diagnosed with CI on the basis of focal neurological defects and brain CT or MRI examinations during their hospitalization from January 1, 2016, to November 1, 2020.

*Hypertension*: patients who were previously diagnosed with hypertension and had received antihypertensive therapy or patients whose systolic pressure ≥ 140 mmHg and/or diastolic pressure ≥ 90 mmHg were found twice during their hospitalization from January 1, 2016, to November 1, 2020.

Long-term use of diuretics and nonsteroidal anti-inflammatory drugs (NSAIDS) was defined as taking the medicine for three months or more.

### 2.3. Statistical Analysis

The continuous data were calculated and expressed as median (min–max) or mean ± standard deviation; the classified variables were represented as the count and percentage. Statistically significant differences were determined using the chi-squared test, one-way analysis of variance, Kruskal-Wallis test, and Bonferroni's multiple comparison test. Pearson (point-biserial) correlation analysis was performed among sUA levels, demographic characteristics, and clinical and laboratory variables. Variables with *p* < 0.05 were involved in the multivariate linear regression model. All data were statistically analyzed using Statistical Package for the Social Sciences (SPSS) software version 21. Two-tailed *p* < 0.05 was considered statistically significant.

## 3. Results

### 3.1. Demographic and Laboratory Variables and Clinical Characteristics among the Three Groups

The information was collected from 3464 patients with gout from January 1, 2016, to November 1, 2020, which included 626 gout+CHD patients, 584 gout+CI patients, and 151 gout+CHD+CI patients. According to the inclusion and exclusion criteria, 753 discharged patients were finally included, including 353 cases of gout+CHD, 298 cases of gout+CI, and 102 cases of gout+CHD+CI. The demographic information, laboratory variables, and clinical characteristics of the subjects are listed in Table 1. Most of the patients were male (gout+CI+CHD: 84 (82.4), gout+CHD: 328 (92.9), and gout+CI: 270 (90.6)). The vast majority of patients are elderly patients (gout+CHD+CI: 76.5 ± 11.8, gout+CHD: 67.8 ± 11.7, and gout+CI: 70.4 ± 11.6). There are statistically significant differences in age, gender, BMI, drinking, long-term use of diuretics, long-term use of NSAIDs, sUA, CRE, TC, TG, LDL, and blood glucose among the gout+CHD group, the gout+CI group, and gout+CHD+CI group (*p* < 0.05), while no significant differences in other variables such as smoking and hypertension were observed (*p* > 0.05) (Table 1).

Next, we compared the characteristics that have significant differences between the three groups in pairs. As shown in Table 2, the gout+CHD and gout+CI+CHD groups, as well as the gout+CI and gout+CI+CHD groups, had statistically significant gender differences (*p* < 0.05); the gout+CI and gout+CHD groups, as well as the gout+CI and gout+CI+CHD groups, had statistically significant differences in drinking and long-term use of diuretics and NSAIDs (*p* < 0.05). Abnormal metabolism plays an important role in gout and cardiovascular and cerebrovascular diseases. Therefore, we also explored the differences in metabolic parameters between groups. There were statistically significant differences in TC and LDL between the gout+CI and gout+CI+CHD groups, as well as the gout+CHD and gout+CI+CHD groups (*p* < 0.05), while no considerable difference was observed between the gout+CI and gout+CHD groups. However, the CRE and blood glucose were markedly decreased in gout+CI patients when compared with gout+CHD patients (*p* < 0.05) (Table 3). The comparison between groups found that there was no difference in BMI between any two groups, but sUA was significantly lower in the gout+CI patients than in the gout+CHD group (*p* < 0.05) (Table 4). The above results suggest that there are significant differences in the clinical characteristics of gout+CI and gout+CHD. It is necessary to further explore the influencing factors of the sUA level in patients with gout+CI.

### 3.2. Correlation of sUA with Demographic Characteristics, Clinical Characteristics, and Biochemical Variables

The uric acid value of gout+CI was significantly lower than that of gout+CHD patients, but there was no statistical difference between the gout+CI and gout+CI+CHD groups. It is possible that CI has an effect on gout+CI+CHD patients. In order to explore the reason why the sUA level of the gout+CI patients is statistically lower than that of the gout+CHD patients, we conducted an analysis of factors related to the sUA level. As shown in Table 5, sUA levels in the gout+CI patients was negatively correlated with age and gender and positively correlated with smoking, CRE, and TG (*p* < 0.05). In contrast, the levels of sUA in the gout+CHD group and the gout+CI+CHD group were only positively correlated with CRE (*p* < 0.05). These results reveal that the influencing factors of UA in patients with gout+CI are significantly different from those of gout+CHD.

### 3.3. Multiple Linear Regression of sUA Factors Related to Gout+CI Patients

The above results show that multiple factors are significantly related to sUA in patients with gout+CI. We further used multiple linear regression to analyze the influencing factors of UA in patients with gout+CI. After excluding nonstatistically significant factors, age, gender, smoking, CRE, and TG were included in the multiple linear regression model, and Pearson correlation analysis was performed on the various factors of the sUA level. The multiple linear regression model was statistically significant (*F* = 15.714, *p* < 0.001). As is shown in Table 6, smoking (*B* = 28.887, *p* = 0.026), CRE (*B* = 1.277, *p* ≤ 0.001), and TG (*B* = 15.993, *p* = 0.005) showed a positive correlation with sUA levels, while age (*B* = −1.392, *p* = 0.013) was negatively correlated with sUA levels. However, gender was not significantly associated with sUA levels (*p* = 0.595). Among the relevant factors, CRE had the most significant influence on sUA levels (beta = 0.380).

## 4. Discussion

Here, we conducted a cross-sectional study on the gout+CHD group, gout+CI group, and gout+CHD+CI patients to explore the characteristics of gout+CI patients and the factors that affect uric acid in these patients. The data showed significant differences in age, drinking, hypertension, long-term use of diuretics and NSAIDs, sUA, CRE, and blood glucose in patients with gout+CHD and gout+CI. The sUA level of the gout+CI group was lower than that of the gout+CHD group. In addition, there are differences in factors affecting uric acid levels between gout+CHD patients and gout+CI patients.

In the gout+CI group, there were significant correlations between age, smoking, CRE, and TG and sUA, while a negative correlation between age and sUA was observed, which may be because the population we included is mainly elderly men (70.4 ± 11.6). Young men are more likely to have an unhealthy lifestyle, including excessive alcohol, sugary drinks, and excessive intake of high-purine foods, such as meat and seafood. It has shown that unhealthy eating habits will gradually improve with age, which may lead to a decrease in sUA levels [9, 10], but this is contrary to some other studies [11, 12]. This may indicate that the younger the patient, the more timely sUA should be reduced. Women often have low sUA levels and rarely get gout. However, there is no significant correlation between gender and sUA level in our study, which may be because the gout women included in the group are all elderly women who have passed menopause and have lost estrogen protection. However, in the group comparison and Spearman correlation analysis, there is a statistical difference in gender, which is consistent with a previous study [13].

Among these related factors, CRE has the most significant impact on sUA levels. Two-thirds of sUA are excreted through the kidneys, and one-third is excreted through the intestines. sUA is filtered in the glomerulus and then reabsorbed and secreted in the renal tubules, but about 90% of the excreted UA is reabsorbed [14–16]. The renal excretion of sUA depends on specific transporters, including URAT1 (uric acid transporter 1), GLUT9 (glucose transporter 9), and BCRP (breast cancer resistance protein). Therefore, impaired renal function may lead to an increase in sUA, which may be a decrease in glomerular filtration or a decrease in renal tubular resecretion [17], accompanied by an increase in CRE. Adversely, It has been proved that the level of sUA is closely related to the progression of nephropathy [18, 19], which may be related to sUA causing kidney damage by stimulating the adrenaline-angiotensin system and promoting the proliferation of vascular smooth muscle cells [20, 21], as well as high uric acid. Symptoms of hyperemia can affect renal hemodynamics and cause glomerular perfusion disorders [22]. In addition, sUA crystals may damage the renal tubules through inflammasome [23], resulting in a decrease in the glomerular filtration rate and an increase in creatinine levels. In this study, we only collected data on patients with gout. Therefore, these patients may suffer a large amount of inflammatory burden and potential kidney damage, which is consistent with the results of this study.

High levels of sUA may cause proinflammatory endocrine imbalance in adipose tissue [24]. Animal experiments have shown that high sUA caused by excessive intake of fructose-containing beverages can lead to the accumulation of triglycerides, and it has also been shown that sUA levels are positively correlated with TG [25, 26]. In addition, the relationship between TG and sUA levels has been demonstrated to be genetically related [27]. In this study, we also found that there is a positive correlation between sUA and TG in gout+CI patients, and the related mechanisms need to be further explored. Previous studies have found that after adjusting the confounding factors that affect the sUA level, the sUA level of the smoking group was significantly lower than that of the nonsmoking group [28]. However, it has been reported that female smokers have a positive correlation with sUA levels, while no significant correlation has been found between male smokers and sUA levels [13]. Conversely, another study demonstrated that male smokers were negatively correlated with sUA levels, while female smokers did not find a significant correlation with sUA levels [29]. The related mechanisms remain elusive, since sUA has not only prooxidative activity but also antioxidant effect [30, 31], which can act as an antioxidant against oxidative stress induced by smoking. Our results show that smoking is positively correlated with sUA levels, and the reasons for the different results may be related to the differences in race, diet, environmental factors, and genetics of the population included in each study.

## 5. Conclusions

Here, we found that the level of sUA in patients with gout+CI was significantly positively correlated with smoking, CRE, and TG and negatively correlated with age. These data may help us strengthen the monitoring and management of gout patients: for the smoking, high CRE, high TG, and younger gout patients, sooner sUA-lowering treatment should be started. Actually, smoking and triglyceride are both risk factors for CI. The main limitation of this study is that cross-sectional studies cannot provide evidence of causality. At the same time, this study has the following shortcomings: this study only included Chinese people, which limits our ability to extend the results to other races; this study did not include asymptomatic hyperuricemia patients, which limits the generality of our study; in addition, only a small number of women with gout are included, and the results may be controversial if they are grouped by gender. Therefore, further prospective research and basic research are needed to explore the relationship between gout and CI and the factors that affect the uric acid level of gout+CI.

## Figures and Tables

**Table 1:** Comparisons of variables in the gout+CI group, the gout+CHD group, and the gout+CI+CHD group.

Characteristics	Gout+CI+CHD (*n* = 102)	Gout+CHD (*n* = 353)	Gout+CI (*n* = 298)	*p* value
Age (year)	76.5 ± 11.8	67.8 ± 11.7	70.4 ± 11.6	<0.001^2^
Gender, male, *n* (%)	84 (82.4)	328 (92.9)	270 (90.6)	0.006^1^
BMI (kg/m^2^)	23.9 ± 3.7	24.7 ± 3.6	24.1 ± 3.4	0.027^2^
Underweight (<18.5), *n* (%)	5 (4.9)	15 (4.3)	14 (4.7)	
Normal weight (18.5~23.9), *n* (%)	45 (44.1)	136 (38.5)	136 (45.6)	
Overweight (24.0~27.9), *n* (%)	41 (40.2)	147 (41.6)	110 (36.9)	
Obesity (≥28), *n* (%)	11 (10.8)	55 (15.6)	38 (12.8)	
Smoking, *n* (%)	35 (34.3)	162 (45.9)	128 (43.0)	0.115^1^
Drinking, *n* (%)	21 (20.6)	98 (27.8)	123 (58.7)	<0.001^1^
Hypertension, *n* (%)	49 (48.0)	155 (43.9)	158 (53.0)	0.068^1^
Long-term use of diuretics, *n* (%)	3 (2.9)	10 (2.8)	39 (13.1)	<0.001^1^
Long-term use of NSAIDs, *n* (%)	27 (26.5)	84 (23.8)	25 (8.4)	<0.001^1^
sUA (*μ*mol/L)	454.1 ± 133.4	497.4 ± 132.8	465.5 ± 118.2	<0.001^2^
CRE (mmol/L)	94.5 (78.0-117.3)	99.0 (79.0-125.0)	91.5 (74.8-114.3)	0.016^3^
TC (mmol/L)	4.0 ± 1.2	4.2 (3.5-5.0)	4.3 (3.5-5.1)	0.003^3^
TG (mmol/L)	1.3 (0.9-1.9)	1.5 (1.1-2.0)	1.4 (1.0-1.9)	0.041^3^
LDL (mmol/L)	2.1 (1.4-2.9)	2.2 (1.8-2.9)	2.4 (1.9-3.0)	0.004^3^
Blood glucose (mmol/L)	5.7 (4.7-7.0)	5.8 (5.0-7.5)	5.5 (4.8-7.0)	0.011^3^

BMI: body mass index; sUA: serum uric acid; CRE: creatinine; TC: total cholesterol; TG: triglycerides; LDL: low-density lipoprotein; gout+CI+CHD: gout with cerebral infarction and coronary heart disease group; gout+CHD: gout with coronary heart disease group; gout+CI: gout with cerebral infarction group. Data with a normal distribution were represented as the mean ± standard deviation, and data with abnormal distribution were represented as median (interquartile range). The classified variables were represented as the count and percentage. ^1^Chi-squared test, ^2^one-way analysis of variance, and ^3^Kruskal-Wallis test were used for the significance of difference between three groups.

**Table 2:** Comparison of classified variables within groups.

	Gender, male		Drinking		Smoking		Long-term use of diuretics		Long-term use of NSAIDs		Hypertension	
	*χ* ^2^	*p* value	*χ* ^2^	*p* value	*χ* ^2^	*p* value	*χ* ^2^	*p* value	*χ* ^2^	*p* value	*χ* ^2^	*p* value
Gout+CI vs. gout+CHD	1.157	0.282	13.159	<0.001	0.565	0.452	8.324	0.004	21.966	<0.001	5.373	0.02
Gout+CHD vs. gout+CI+CHD	10.322	0.001	2.109	0.146	4.321	0.038	0.003	0.954	0.307	0.58	0.546	0.46
Gout+CI vs. gout+CI+CHD	5.083	0.024	14.115	<0.001	2.349	0.125	24.412	<0.001	27.515	<0.001	0.755	0.385

*p* value of significance assessed by the chi^2^ test (categorical variables) and Bonferroni correction.

**Table 3:** Comparison of nonnormal distribution or unequal comparison of continuous variables within groups.

	TC	CRE	TG	LDL	Blood glucose
Gout+CI *vs.* gout+CHD, *p* value	0.671	0.013	0.097	0.614	0.016
Gout+CHD *vs.* gout+CI+CHD, *p* value	0.025	0.786	0.058	0.041	0.162
Gout+CI *vs.* gout+CI+CHD, *p* value	0.002	1	0.746	0.003	1

CRE: creatinine; TC: total cholesterol; TG: triglycerides; LDL: low-density lipoprotein. *p* value of significance assessed by Kruskal-Wallis' test (continuous variables) and Bonferroni correction.

**Table 4:** Comparison of normal distribution and equal comparison of continuous variables within groups.

	sUA	BMI	Age
Gout+CI vs. gout+CHD, *p* value	0.005	0.076	0.018
Gout+CHD vs. gout+CI+CHD, *p* value	0.008	0.107	<0.001
Gout+CI vs. gout+CI+CHD, *p* value	1	1	<0.001
*F*	7.306	3.579	21.884
*p*	<0.001	0.028	<0.001

sUA: serum uric acid; BMI: body mass index. *p* value of significance assessed by one-way analysis of variance (continuous variables) and Bonferroni correction.

**Table 5:** Correlation of sUA with demographic characteristics, clinical characteristics, and laboratory variables.

Variables	Gout+CI+CHD		Gout+CHD		Gout+CI	
	Pearson coefficient	*p* value	Pearson coefficient	*p* value	Pearson coefficient	*p* value
Age (year)	-0.094	0.350	-0.050	0.924	-0.127	0.028
Gender, male, *n* (%)	-0.162	0.103	-0.073	0.173	-0.126	0.029
BMI (kg/m^2^)	0.019	0.850	0.064	0.233	0.068	0.243
Smoking, *n* (%)	0.133	0.182	0.033	0.537	0.209	<0.001
Drinking, *n* (%)	0.161	0.107	0.052	0.332	0.089	0.125
Hypertension, *n* (%)	-0.016	0.876	0.026	0.620	-0.075	0.198
Long-term use of diuretics, *n* (%)	-0.038	0.706	0.055	0.306	0.073	0.210
Long-term use of NSAIDs, *n* (%)	0.104	0.299	0.075	0.160	-0.060	0.300
CRE (mmol/L)	0.198	0.046	0.230	<0.001	0.369	<0.001
TC (mmol/L)	-0.058	0.564	-0.029	0.592	0.036	0.532
TG (mmol/L)	-0.037	0.709	0.037	0.489	0.169	0.003
LDL-C (mmol/L)	0.110	0.272	-0.035	0.509	-0.004	0.947
Blood glucose (mmol/L)	0.065	0.517	0.088	0.100	0.013	0.826

Spearman correlation analysis was used for the significance of difference.

**Table 6:** Multiple linear regression of factors related to sUA in the gout+CI group.

Variables	Unstandardized coefficient		Standardized coefficient	*T*	*p*	VIF
	*B*	SE	Beta			
Age	-1.392	0.557	-0.137	-2.501	0.013	1.114
Gender, male	11.595	21.803	0.029	0.532	0.595	1.077
Smoking	28.887	12.937	0.121	2.233	0.026	1.091
CRE	1.277	0.181	0.380	7.051	<0.001	1.075
TG	15.993	5.706	0.150	2.803	0.005	1.065

## Data Availability

The data used to support the findings of this study are available from the corresponding author upon request.

## References

[1] Primatesta P., Plana E., Rothenbacher D. (2011). Gout treatment and comorbidities: a retrospective cohort study in a large US managed care population. *BMC Musculoskeletal Disorders*.

[2] Lee S. Y., Park W., Suh Y. J. (2019). Association of serum uric acid with cardiovascular disease risk scores in Koreans. *International Journal of Environmental Research and Public Health*.

[3] Huang W. S., Lin C. L., Tsai C. H., Chang K. H. (2020). Association of gout with CAD and effect of antigout therapy on CVD risk among gout patients. *Journal of Investigative Medicine*.

[4] Lai X., Yang L., Légaré S. (2016). Dose-response relationship between serum uric acid levels and risk of incident coronary heart disease in the Dongfeng-Tongji cohort. *International Journal of Cardiology*.

[5] Zhang B., Yang N., Lin S. P., Zhang F. (2017). Suitable concentrations of uric acid can reduce cell death in models of OGD and cerebral ischemia-reperfusion injury. *Cellular and Molecular Neurobiology*.

[6] Hozawa A., Folsom A. R., Ibrahim H., Nieto F. J., Rosamond W. D., Shahar E. (2006). Serum uric acid and risk of ischemic stroke: the ARIC study. *Atherosclerosis*.

[7] Kim S. Y., Guevara J. P., Kim K. M., Choi H. K., Heitjan D. F., Albert D. A. (2009). Hyperuricemia and risk of stroke: a systematic review and meta-analysis. *Arthritis and Rheumatism*.

[8] Neogi T., Jansen T. L., Dalbeth N. (2015). 2015 gout classification criteria: an American College of Rheumatology/European League Against Rheumatism collaborative initiative. *Annals of the Rheumatic Diseases*.

[9] Kim I. Y., Han K. D., Kim D. H. (2019). Women with metabolic syndrome and general obesity are at a higher risk for significant hyperuricemia compared to men. *Journal of Clinical Medicine*.

[10] Cao J., Wang C., Zhang G. (2017). Incidence and simple prediction model of hyperuricemia for urban Han Chinese adults: a prospective cohort study. *International Journal of Environmental Research and Public Health*.

[11] Kapetanovic M. C., Hameed M., Turkiewicz A. (2016). Prevalence and incidence of gout in southern Sweden from the socioeconomic perspective. *RMD Open*.

[12] Rai S. K., Aviña-Zubieta J. A., McCormick N. (2017). The rising prevalence and incidence of gout in British Columbia, Canada: population-based trends from 2000 to 2012. *Seminars in Arthritis and Rheumatism*.

[13] Kim S. K., Choe J. Y. (2019). Association between smoking and serum uric acid in Korean population: data from the seventh Korea national health and nutrition examination survey 2016. *Medicine (Baltimore)*.

[14] Johnson R. J., Bakris G. L., Borghi C. (2018). Hyperuricemia, acute and chronic kidney disease, hypertension, and cardiovascular disease: report of a scientific workshop organized by the National Kidney Foundation. *American Journal of Kidney Diseases*.

[15] George C., Minter D. A. (2020). Hyperuricemia. *StatPearls*.

[16] Keenan R. T. (2020). The biology of urate. *Seminars in Arthritis and Rheumatism*.

[17] Barkas F., Elisaf M., Liberopoulos E., Kalaitzidis R., Liamis G. (2018). Uric acid and incident chronic kidney disease in dyslipidemic individuals. *Current Medical Research and Opinion*.

[18] Weiner D. E., Tighiouart H., Elsayed E. F., Griffith J. L., Salem D. N., Levey A. S. (2008). Uric acid and incident kidney disease in the community. *Journal of the American Society of Nephrology*.

[19] Sellmayr M., Hernandez Petzsche M. R., Ma Q. (2020). Only hyperuricemia with crystalluria, but not asymptomatic hyperuricemia, drives progression of chronic kidney disease. *Journal of the American Society of Nephrology*.

[20] Perlstein T. S., Gumieniak O., Hopkins P. N. (2004). Uric acid and the state of the intrarenal renin-angiotensin system in humans. *Kidney International*.

[21] Rao G. N., Corson M. A., Berk B. C. (1991). Uric acid stimulates vascular smooth muscle cell proliferation by increasing platelet-derived growth factor A-chain expression. *The Journal of Biological Chemistry*.

[22] Uedono H., Tsuda A., Ishimura E. (2015). Relationship between serum uric acid levels and intrarenal hemodynamic parameters. *Kidney & Blood Pressure Research*.

[23] Braga T. T., Foresto-Neto O., Camara N. O. S. (2020). The role of uric acid in inflammasome-mediated kidney injury. *Current Opinion in Nephrology and Hypertension*.

[24] Baldwin W., McRae S., Marek G. (2011). Hyperuricemia as a mediator of the proinflammatory endocrine imbalance in the adipose tissue in a murine model of the metabolic syndrome. *Diabetes*.

[25] Tapia E., Cristóbal M., García-Arroyo F. E. (2013). Synergistic effect of uricase blockade plus physiological amounts of fructose-glucose on glomerular hypertension and oxidative stress in rats. *American Journal of Physiology. Renal Physiology*.

[26] Ali N., Rahman S., Islam S. (2019). The relationship between serum uric acid and lipid profile in Bangladeshi adults. *BMC Cardiovascular Disorders*.

[27] Moriwaki Y., Yamamoto T., Takahashi S., Tsutsumi Z., Higashino K. (1995). Apolipoprotein E phenotypes in patients with gout: relation with hypertriglyceridaemia. *Annals of the Rheumatic Diseases*.

[28] Hanna B. E., Hamed J. M., Touhala L. M. (2008). Serum uric acid in smokers. *Oman Medical Journal*.

[29] Gee Teng G., Pan A., Yuan J. M., Koh W. P. (2016). Cigarette smoking and the risk of incident gout in a prospective cohort study. *Arthritis care & research*.

[30] Sautin Y. Y., Johnson R. J. (2008). Uric acid: the oxidant-antioxidant paradox. *Nucleosides, Nucleotides & Nucleic Acids*.

[31] Waring W. S., Webb D. J., Maxwell S. R. (2001). Systemic uric acid administration increases serum antioxidant capacity in healthy volunteers. *Journal of Cardiovascular Pharmacology*.

